# Concurrent Appendicitis and Meckel’s Diverticulitis Presenting With Small Bowel Obstruction in an Adult: A Case Report

**DOI:** 10.7759/cureus.67000

**Published:** 2024-08-16

**Authors:** Killian J Llewellyn, Michaela Knaggs, Thomas Serena, Jeffrey R Gerken

**Affiliations:** 1 General Surgery, Corewell Health Farmington Hills, Farmington Hills, USA

**Keywords:** community, laparoscopy, small bowel obstruction, meckel's diverticulum, appendicitis

## Abstract

We present a case of multiple abdominal pathologies occurring simultaneously, which emphasizes the importance of keeping a broad differential and evaluating each diagnosis. A 33-year-old female presented with abdominal pain, nausea, and vomiting. Her workup included computerized tomography which demonstrated acute appendicitis with concern for a closed-loop bowel obstruction. She was offered diagnostic laparoscopy with anticipation of laparoscopic appendectomy and further evaluation for the source of the bowel obstruction. At the time of surgery, a Meckel’s diverticulum with acute diverticulitis was identified, in addition to an inflamed appendix. A small band near the base of the Meckel’s diverticulum was found and divided. The appendix was treated with a laparoscopic appendectomy and the Meckel's diverticulum was resected. She did well in recovery and continued to do well at her follow-up appointment. This case emphasized the importance of a thorough evaluation of a patient’s differential diagnosis, as it is possible for multiple pathologies to occur simultaneously.

## Introduction

Acute appendicitis is a common surgical emergency. Meckel's diverticulum, a congenital anatomical variant involving the small bowel, has the potential to mimic acute appendicitis clinically. With the potential for similar presentations, a Meckel’s diverticulum should be included in the differential for presentations consistent with acute appendicitis. A Meckel’s diverticulum may be found at the time of laparoscopic appendectomy when the appendix has a normal appearance intraoperatively. We present a case of concurrent acute appendicitis and Meckel’s diverticulitis presenting with a small bowel obstruction in an adult managed in the community setting with laparoscopy. 

Meckel’s diverticulum has occasionally been reported to be associated with other pathologies. Mudatsiks et al. described a case of appendicitis with a concurrent Meckel’s diverticulum that contained a carcinoid tumor, which was managed with an open surgical approach [1]. Mohiuddin et al. describe a case of Meckel’s diverticulum presenting as acute appendicitis, but diagnostic laparoscopy revealed a small bowel obstruction with a normal appendix [2]. In this case report, we present the simultaneous occurrence of both acute appendicitis and a Meckel’s diverticulum causing small bowel obstruction. Diverticulitis of a Meckel’s diverticulum occurs in approximately 20% of patients [3]; however, to our knowledge, there is no previously reported literature of concurrent appendicitis with Meckel’s diverticulitis presenting as a small bowel obstruction in an adult. There have been sparse reports of the combination of acute appendicitis with concurrent Meckel's diverticulum leading to obstruction, but this has only been previously described in the pediatric population [4].

## Case presentation

A 33-year-old female presented with one day of periumbilical abdominal pain. This was associated with nausea, vomiting, and diarrhea. She also was not passing flatus. The patient denied a history of similar pain or symptoms. There was no prior personal or family history of colon cancer, ulcerative disease, or Crohn’s disease. Her surgical history was significant for laparoscopic cholecystectomy. 

The patient was hemodynamically stable and afebrile on initial presentation. Her abdominal exam was significant for diffuse tenderness to percussion, involuntary guarding, and a positive McBurney’s sign. No rebound tenderness was noted. Laboratory studies obtained were unremarkable apart from a mildly elevated white blood cell count at 12.5 x 10^3^. 

Computed tomography of the abdomen and pelvis with intravenous (IV) contrast demonstrated a mildly dilated distal small bowel loop with surrounding fat stranding. It was unclear if this dilated loop represented Meckel's diverticulum versus a focal closed loop obstruction. Additionally, the appendix was 9 mm in diameter and with mild periappendiceal fat stranding. There was no evidence of intra-abdominal abscess, perforation, or pneumoperitoneum. 

**Figure 1:**
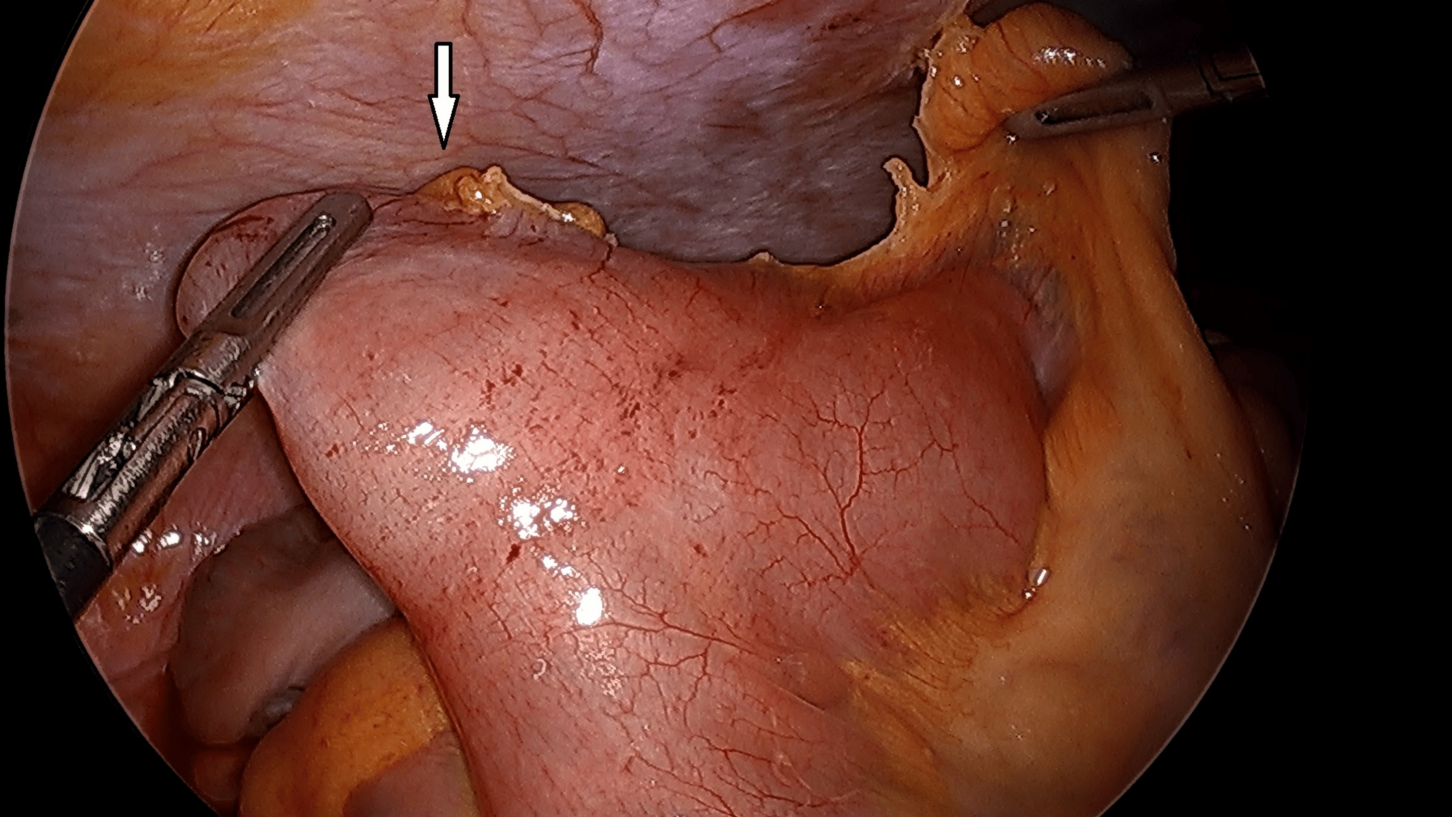
Meckel's diverticulum held in a grasper

It was recommended that the patient be taken for diagnostic laparoscopy with possible intervention for suspected acute appendicitis as well as possible closed-loop small bowel obstruction. She was agreeable to surgical intervention and was taken to the operating room. A diagnostic laparoscopy was performed. The appendix was identified and was noted to be acutely inflamed. A vessel sealer device was utilized to mobilize and cauterize the mesoappendix, and the appendix was stapled at the base. Following this, attention was turned to the small bowel. Approximately 60 cm proximal to the cecum, there was an obvious enlarged and inflamed Meckel's diverticulum, as seen in Figure 1. 

**Figure 2:**
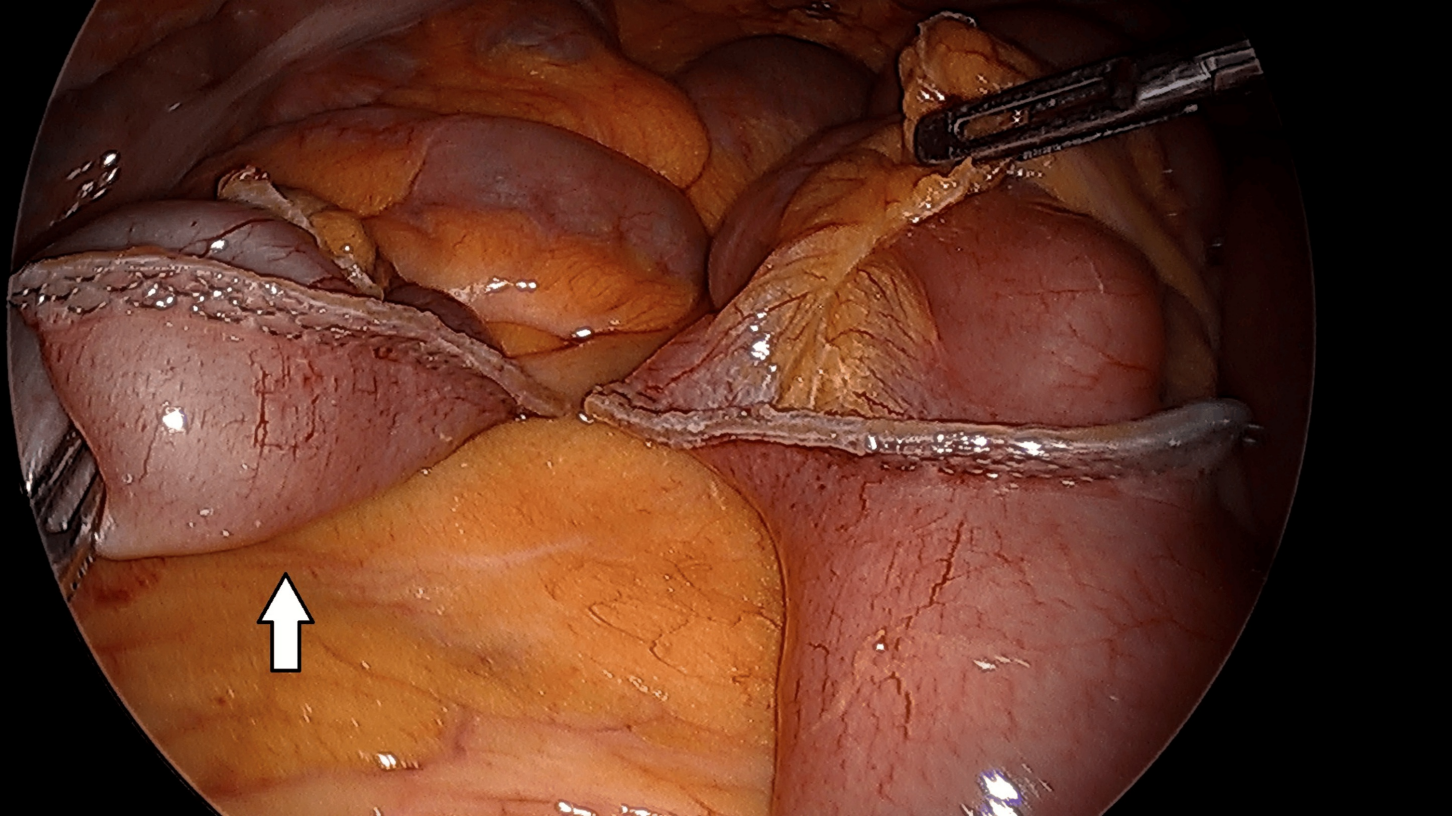
Meckel's diverticulum divided at its base (white arrow)

A small band was found near the base of the diverticulum, which appeared to be congenital in nature. This was divided with hook electrocautery. The decision was made to perform a diverticulectomy. The patient had no history of any type of gastrointestinal bleed, and thus, the decision was made to perform a simple diverticulectomy. The diverticulum was elevated, and an endoscopic stapler was placed across the base of the diverticulum to perform a diverticulectomy for horizontal closure across the bowel (Figure 2). 

This was done with no tension on the bowel. Following this, the entirety of the small bowel was again run from the cecum to the ligament of Treitz with no additional findings. There was turbid fluid in the pelvis which was removed with suction.

She was observed overnight. On the morning after surgery, the patient’s abdominal pain had improved. The patient was afebrile and without leukocytosis. She had tolerated a clear liquid diet overnight and progressed to a regular diet the morning following her procedure. Her Foley catheter was removed. She was discharged home the afternoon after surgery in stable condition. 

Pathology was reviewed with two separate entities noted. The appendix specimen was found to have acute focal appendicitis. The small bowel specimen was described in the pathology report as benign small bowel with an edematous wall and centrally hemorrhagic. 

The patient followed up in the office three weeks after her surgery. At follow-up, the patient’s abdominal pain had subsided. She was tolerating a regular diet and having no issues with hematochezia, melena, or constipation.

## Discussion

We present the rare concurrence of two pathologies occurring at the same time. While acute appendicitis is a common pathology, it is important to keep a symptomatic Meckel’s diverticulum as part of the differential diagnosis. When a normal-appearing appendix is observed surgically and the patient displays symptoms of acute appendicitis, it should prompt further exploration to identify the source of the patient’s symptoms. However, as demonstrated in this patient, it is important to be aware that multiple pathologies can occur at the same time.

In this patient’s case, Meckel’s diverticulum was noted to be associated with a small bowel obstruction. Preoperative imaging was concerning for a closed-loop bowel obstruction, and at the time of operation, a Meckel’s diverticulum with a small band was visualized, appearing to be the source of the obstruction. A review by Hansen and Søreide found that intestinal obstruction is the most common presentation for a symptomatic Meckel’s diverticula, followed by gastrointestinal bleeding and inflammation [5]. Of note, more than 50% of symptomatic Meckel’s diverticula occur in populations less than 10 years old [3]. Shademan and Tappouni note that a Meckel’s diverticulum itself can be misdiagnosed as acute appendicitis with cross-sectional imaging [6]. This demonstrates one of the limitations of computerized tomography when evaluating the small bowel where a diagnostic laparoscopy can provide additional information. Given that the appendix was inflamed intraoperatively and on pathological evaluation, this patient’s acute appendicitis was diagnosed correctly via cross-sectional imaging. The area seen on imaging concerning a closed-loop bowel obstruction proved to be a Meckel's diverticulum.

We managed this case with a minimally invasive approach, utilizing laparoscopy as a starting approach to confirm the diagnosis. The acute appendicitis was managed with laparoscopic appendectomy, while the Meckel’s diverticulum and associated bowel obstruction were resolved with lysis of an adhesive band and diverticulectomy. Had there been more extensive pathology, the team was prepared to convert to a laparotomy. Laparoscopic approaches for Meckel’s diverticulum have been noted to be an effective approach for management [5]. A Meckel’s diverticulum has the potential to harbor ectopic tissue, such as gastric or pancreatic, that can cause ulceration of the mucosa leading to bleeding. This can occur opposite to the diverticulum, leaving a bleeding ulceration behind if a diverticulectomy alone is performed without resection of the bowel. We elected to perform a diverticulectomy as opposed to a bowel resection and anastomosis as the patient denied any history of gastrointestinal bleeding.

## Conclusions

We present a rare case of the simultaneous occurrence of multiple etiologies often included in the differential for abdominal pain: acute appendicitis, Meckel’s diverticulum, and small bowel obstruction occurring in the adult population. This patient’s pathology was able to be managed in the community setting with a minimally invasive approach via laparoscopic appendectomy and Meckel’s diverticulectomy with lysis of an obstructing band. This case demonstrates the importance of maintaining a high index of suspicion for less common pathologies. The case also serves as a reminder that multiple pathologic processes may potentially be contributing to a patient’s clinical presentation.
